# Transcriptomic analysis identifies shared biological foundations between ischemic stroke and Alzheimer’s disease

**DOI:** 10.3389/fnins.2022.1008752

**Published:** 2022-11-18

**Authors:** Wenhao Liu, Mengyao Wan, Yinchao Shi, Xin-Zhuang Yang

**Affiliations:** ^1^Department of Neurology, Peking Union Medical College Hospital, Chinese Academy of Medical Sciences and Peking Union Medical College, Beijing, China; ^2^Department of Biochemistry and Molecular Biology, Institute of Basic Medical Sciences, Chinese Academy of Medical Sciences and School of Basic Medicine, Peking Union Medical College, Beijing, China; ^3^Medical Research Center, State Key Laboratory of Complex Severe and Rare Diseases, Peking Union Medical College Hospital, Chinese Academy of Medical Sciences and Peking Union Medical College, Beijing, China

**Keywords:** transcriptomic analysis, ischemic stroke, Alzheimer’s disease, shared biological dimensions, immune system

## Abstract

**Aim:**

Alzheimer’s disease (AD) and ischemic stroke (IS), two major neurological diseases, are suggested to be associated in clinical and pathophysiological levels. Previous studies have provided some insights into the possible genetic mechanisms behind the correlation between AD and IS, but this issue is still not clear. We implemented transcriptomic analysis to detect common hub genes and pathways to help promote the understanding of this issue.

**Materials and methods:**

Four gene expression profiling datasets (GSE16561, GSE58294, GSE63060, and GSE63061) of peripheral whole blood, which contain 108 IS samples, 284 AD samples, and 285 matched controls, were employed to detect differentially expressed genes (DEGs) for AD and IS, which were further analyzed for shared biological pathways, candidate drugs, and transcription factors. Protein-protein interaction (PPI) network and drug-target interaction analysis were applied to identify hub genes and drug targets, respectively. Result verification was done with other independent datasets (GSE37587, GSE46480, and GSE140829). The difference in proportions of various immune cells in the peripheral blood of AD and IS patients were evaluated using CIBERSORT.

**Results:**

We identified 74 DEGs and 18 biological processes with statistical significance shared by AD and IS, 9 of which were immune-related pathways. Five hub genes scored high in the topological analysis of the PPI network, and we also found eight drug target genes and candidate drugs which were associated with AD and IS. As for immunological changes, an increase in the proportion of M0 macrophages was found in the peripheral circulation of both AD and IS patients, and *SOD1* expression was significantly correlated with this change.

**Conclusion:**

Collectively, the common DEGs and shared pathways found in this study suggest a potential shared etiology between AD and IS, behind which immune system, particularly the M0 macrophage elevation, might have important roles. While, the shared hub genes, potential therapeutic gene targets and drugs reported in this study provide promising treatment strategies for AD and IS.

## Introduction

Alzheimer’s disease (AD), one of the foremost neurodegenerative diseases, is characterized by memory loss and gradual impairment in language, praxis, and other aspects of cognition, leading to dementia. It is acknowledged that genetic and environmental factors interact with each other during the onset and development of AD (Wicinski et al., 2019). Ischemic stroke (IS) is a major public health problem with high prevalence and disability and mortality rate (Virani et al., 2020). The pathogenesis behind is heterogenous, including atherosclerosis, disturbance in blood regulation, genetic disorders, etc. In recent years, numerous studies have explored the loci and genes associated with these two complicated diseases (Lucke-Wold et al., 2015; Cui et al., 2018; Wang et al., 2021). Despite the seemingly different etiopathogenesis, many clinical studies have indicated potential association between AD and IS at multiple levels (Zhou et al., 2015; Wang et al., 2020, 2021; Pinho et al., 2021; Zhang et al., 2021). Firstly, the coexistence of these two diseases is more frequent than by chance, as a population-based study in 2013 showed that the prevalence of IS was significantly higher in AD patients (Iadecola, 2016). Besides, AD and IS share some common risk factors such as old age, obesity, hypertension, and stroke is known to advance AD development (Breijyeh and Karaman, 2020).

Furthermore, with the progress in sequencing technology, increasing research is exploring the genetic and molecular mechanisms behind these diseases. Studies showed that *APOE* ε*4*, a verified allele related to AD, increased concomitance of AD and IS, and presented a positive dose-response association with the IS (Khan et al., 2013). The amyloid-beta (Aβ) deposition in brain parenchyma and vessels is the pathology for AD and cerebral amyloid angiography, respectively, while the latter is one of the causes for young-onset and recurrent IS (Vijayan and Reddy, 2016; Dong et al., 2018). Mutation in genes influencing Aβ (e.g., *PSEN1*, *APP*) formation could be the causes for both AD and IS (Dong et al., 2018). The genome-wide association study (GWAS) is suitable for exploring the comprehensive effects of genetic factors behind complicated diseases. Cui et al. (2018) summarized GWAS datasets and discovered several shared novel functional pathways linking AD and IS, including immune process and signaling transduction. Wei et al. (2019) found that SNPs previously shown to function in immune system might underlie the common pathogenesis for AD and IS. Our previous studies demonstrated post-IS inflammatory reactions including the differentiation and activation of T cells and mononuclear cells, which were also involved in sporadic AD by previous GWASs (Liu W. et al., 2022).

**FIGURE 1:**
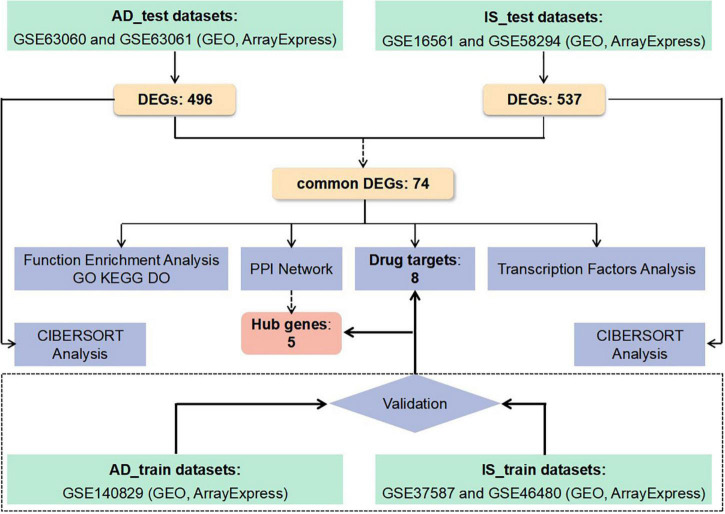
Flowchart of the study. Microarray data from whole peripheral blood of patients with Alzheimer’s disease (AD) or ischemic stroke (IS) were obtained and analysis for differentially expressed genes (DEGs) between patients and healthy controls was performed. Protein-protein interactions network (PPI–Net), drug targets, functional enrichment, and immune infiltration analysis were applied to common DEGs between AD and IS, to explore shared mechanism between these two diseases. Hub genes obtained by PPI-Net analysis and potential drug targets were validated with test dataset of AD and IS.

Despite the above clinical and genetic evidence, it is still very difficult to clarify the pathogenesis underlying the relationship between IS and AD. A genome-wide transcriptome study (GWTS) could provide new perspectives, identifying more relevant pathways. However, few GTWSs have explored the shared biological pathways and transcriptomic changes between IS and AD. Therefore, we applied analyses on the following aspect, protein-protein interaction (PPI) network, functional pathway enrichment, drug targets, transcript factors, and immune infiltration, based on common differentially expressed genes (DEGs) in IS and AD patients. The sequential workflow of our research is presented in Figure 1.

## Materials and methods

### Datasets employed in this study

The microarray datasets used in this study were obtained from the GEO database.^1^ The criteria for retrieval were: (A) samples were from human peripheral whole blood samples, (B) gene expression was profiled, (C) datasets contained both patients and healthy people without a history of stroke nor dementia, (D) all IS patients were clinically diagnosed radiographically (with magnetic resonance imaging or computed tomography), (E) all AD patients were diagnosed according to the National Institute of Neurological and Communicative Disorders and Stroke and the Alzheimer’s Disease and Related Disorders Association criteria. In particular, all the non-IS or non-AD samples included in the datasets were deleted.

To ensure the consistency and completeness of the datasets, we manually identified relevant literature using keywords filters and applied R programming language (version: 4.1.3) for subsequent analysis. Finally, IS datasets [GSE16561 (Barr et al., 2010; O’Connell et al., 2016, 2017) and GSE58294 (Stamova et al., 2014)] and AD datasets [GSE63060 and GSE63061 (Sood et al., 2015)] were included as training sets and were merged, respectively, and batch effects were corrected using the “combat” function in the SVA package (version: 3.38.0). Next, we normalized the merged datasets and adjusted for covariates using the “Normalizebetweenarrays” and “removeBatchEffect” function in the limma package (version: 3.46.0). To validate hub genes and drug targets, we downloaded GSE140829 (Cooper et al., 2018) dataset as validation set for AD, and GSE37587 (Barr et al., 2015), GSE46480 (Issa et al., 2016) datasets for IS which conformed to the above criteria. Table 1 summarizes the included datasets.

**TABLE 1:** All data sets used in this study contain a total of 1,273 samples, among which there were 650 cases and 623 controls.

Data sets (GEO ID)	Data		Sample type	References	Category	Phenotype	GPL
	Case	Control					
GSE16561	39	24	Peripheral blood	Johnston et al., 2018; Kaushal et al., 2015; Khan et al., 2013	Train	Ischemic stroke	GPL570
GSE58294	69	23	Peripheral blood	Lautrup et al., 2019	Train	Ischemic stroke	GPL570
GSE37587	68	0	Peripheral blood	Li et al., 2019	Test	Ischemic stroke	GPL6883
GSE46480	0	98	Peripheral blood	Issa et al., 2016	Test	Control	GPL570
GSE63060	145	104	Peripheral blood	Lee et al., 2010	Train	Alzheimer’s disease*	GPL6947
GSE63061	139	134	Peripheral blood	Lee et al., 2010	Train	Alzheimer’s disease*	GPL10558
GSE140829	190	240	Peripheral blood	Cooper et al., 2018	Test	Alzheimer’s disease*	GPL15988

All samples were collected in the peripheral blood tissue.

*All the MCI samples included in the datasets were deleted, and the Case column only referred to AD samples.

### Identification of differentially expressed genes and functional annotation

To identify differentially expressed genes (DEGs) in peripheral blood samples from AD/IS patients and controls, we performed differential expression analysis using the limma package (version: 3.46.0), controlling for age. The threshold for screening DEGs was | log_2_ FC (fold change)| > 0.5 and false discovery rate (FDR) < 0.01. Common DEGs for AD and IS were then imported to functional annotation.

Enrichment analysis of Gene Ontology (GO) and Disease Ontology (DO) was performed on common DEGs using the clusterprofiler package (version: 3.18.1). Kyoto Encyclopedia of Genes and Genomes (KEGG)^2^ and gene set enrichment analysis (GSEA) were further carried out for common DEGs. The threshold for significance of the above enrichment analysis was set at FDR < 0.05. The background used for biological functional enrichment analysis were genes expressed in any samples of AD and IS in training process.

### Identification and validation of hub genes and drug targets

Protein function prediction is the key step in biology research and drug discovery. In order to explore the functional interaction between the common DEGs of AD and IS, PPI network analysis was adopted, which was provided by the STRINGdb package (version: 2.6.5) with a confidence score of ≥ 0.7 [0,1]. The information of PPI network was further imported into Cytoscape software (version: 3.9.1) for subsequent analyses. We used Cytohubba app^3^ in Cytoscape for network topology analysis to detect hub genes, through which eleven circulation methods were used to score and rank all DEGs.

Drug and Drug_link datasets (Release Version: 5.1.9) were downloaded from the DrugBank database.^4^ The intersection of the common DEGs and drug target genes (DTGs) was then used to generate genes targeted by drugs and potential drugs that might contribute to phenotypes. Validation datasets were further used to examine the robustness of hub genes and drug targets.

### Transcription factors analysis

The common DEGs were imported into Cytospace for network analysis of transcription factors (TFs). RcisTarget package was used to acquire information of TFs and gene targets, and adjusted *P*-value < 0.05 was considered significant. Subsequently, we verified the expression levels of these TFs in validation datasets for AD and IS with *t*-test.

### Immune cell infiltration evaluation

CIBERSORT tool analyzing immune system (version: 0.1.0) was used to generate immune cell profiles for all samples by estimating relative subsets of RNA transcripts. The CIBERSORT resulted in an expression matrix of 22 immune cells in all samples of the training dataset for AD and IS. We then used *t*-test to analyze the differences in immune cell components between AD/IS patients and healthy controls. Finally, Spearman’s correlation analysis was performed between these selected genes (hub genes and drug targets) and significantly differentiated immune cells. The ggplot2 package (version: 3.3.3) and ggpubr package (version: 0.4.0) was used to generate lollipop chart.

## Results

### Identification of separate and common differentially expressed genes of Alzheimer’s disease and ischemic stroke

To identify differentially expressed genes shared by AD and IS, we initially searched the Array Express and NCBI GEO databases for expression data from whole peripheral blood of AD/IS patients and healthy controls. Seven independent studies that met our inclusion criteria were obtained (see Section “Materials and methods” and Tables 1–3).

**TABLE 2:** Clinical characters of the merged IS training data sets.

	Total sample, *N* (%)	Stroke, *N* = 108 (69.7%), *N* (%)	Control, *N* = 47 (30.3%), *N* (%)	Statistics/*df*	*P-*value
Gender (% female)	80 (51.6%)	55 (50.9%)	25 (53.2%)	X^2^ 0.0673/1	0.7953
Age, years, mean ± SD	66.7 ± 16.86	72.6 ± 12.09	58.9 ± 7.51	t −13.90302/135	<0.001
Race (% white)	126 (81.3%)	84 (77.8%)	42 (89.3%)	X^2^ 2.88932/1	0.0892
Hypertension	93 (60.0%)	70 (64.8%)	23 (48.9%)	X^2^ 3.44037/1	0.0636
Diabetes	30 (19.4%)	23 (21.3%)	7 (14.9%)	X^2^ 0.8601/1	0.3537
Dyslipidemia	52 (33.5%)	36 (33.3%)	16 (34.0%)	X^2^ 0.00739/1	0.9333

**TABLE 3:** Clinical characters of the merged AD training data sets.

	Total sample, *N* (%)	AD, *N* = 284 (55.4%), *N* (%)	Control, *N* = 238 (44.6%), *N* (%)	Statistics/*df*	*P-*value
Gender (% female)	327 (62.6%)	184 (64.8%)	143 (60.1%)	X^2^ 1.2247/1	0.2684
Age, years, mean ± SD	70.3 ± 13.07	69.9 ± 13.19	70.5 ± 12.39	t −0.678/513	0.4981
Race (% white)	516 (98.8%)	281 (98.9%)	235 (98.7%)	X^2^ 0.0475/1	0.8275

**FIGURE 2:**
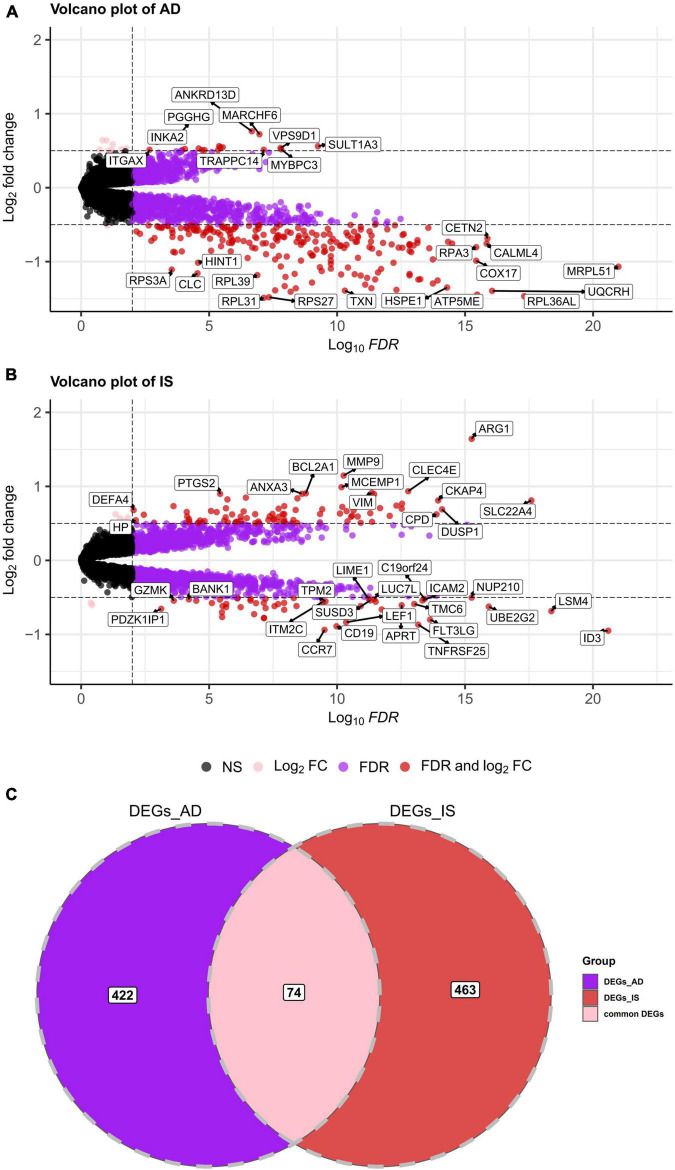
**(A,B)** Volcano plot demonstrating an overview of the differentially expressed genes in AD and IS. The threshold in the volcano plot was –lg(adjusted *P*) > 2 and |log_2_ fold change| > 0.5; red dots indicate significant differentially expressed genes. **(C)** Venn diagram demonstrates the common DEGs of AD and IS. Red, purple, and pink represent significant DEGs of IS, AD, and both AD and IS, respectively.

First, a dataset consisting of 108 IS patients and 47 matched controls was generated by merging two IS datasets: GSE16561 and GSE58294 (Table 2). To ensure data consistency, batch effects were controlled and the different subsets were normalized. The evaluation results showed that data pre-processing was effective and reliable (Supplementary Figures 1, 2). Next, differential analysis of gene expression was performed by controlling age, which is significantly different between two groups (Table 2). Finally, 537 DEGs for IS and 496 DEGs for AD were identified between patients and healthy controls (see Section “Materials and methods” and Figures 2A,B), and we found 74 common DEGs between AD and IS (Figure 2C).

### Functional enrichment analysis of common differentially expressed genes

**FIGURE 3:**
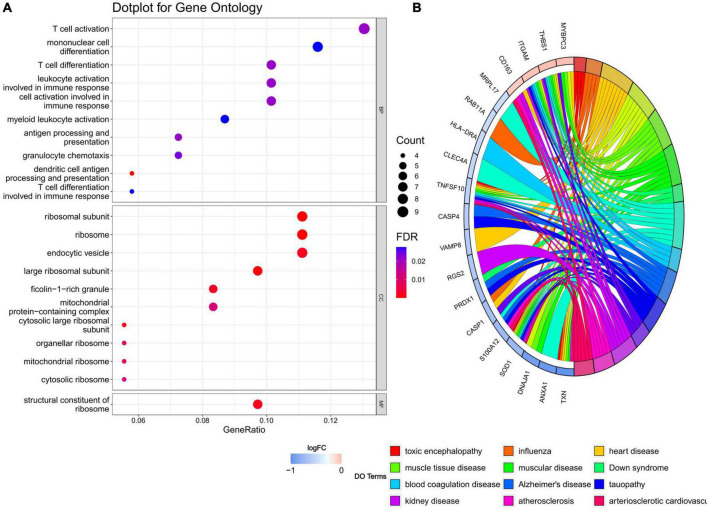
**(A)** GO enrichment analysis, where the horizontal axis represents the proportion of DEGs under the GO term. Top 10 pathways with most significant *P*-value were shown and ordered by gene ratio. BP, biological process; CC, cellular component; MF, molecular function. **(B)** DO enrichment analysis. Chord diagram showed the correlation between diseases and common DEGs, with different colors corresponding to different DO terms.

Gene ontology and disease ontology enrichment analysis were performed to identify the biological pathways and diseases associated with the shared DEGs. GO enrichment analysis explored the biological processes, cellular components, and molecular functions. For biological processes, 18 pathways achieved statistical significance, including antigen processing and presentation, T cell differentiation and activation, cell activation involved in immune responses and mononuclear cell differentiation and so on, half of which were noticeably associated with immunological changes. While for cellular components, ribosome, endocytic vesicle, ficolin-1-rich granule, mitochondrial protein-containing complex, and vesicle membrane were involved. The molecular function significantly associated with common DEGs was structural constituent of ribosome. When performing DO analysis, Alzheimer’s disease, tauopathy, atherosclerosis, and heart disease were related with common DEGs. More information for GO and DO enrichment analysis is presented in Figure 3.

Furthermore, the GSEA results demonstrated that the enriched molecular pathways related to DEGs were cellular responses to stress and stimuli, innate immune system, translation, and neutrophil degranulation (Supplementary Figure 3). These results were consistent with those in GO enrichment analysis considering innate immune involvement. These results provide evidence that immune-related biological processes might play important roles in the connection of AD and IS.

### Identification of hub genes and drug targets

**FIGURE 4:**
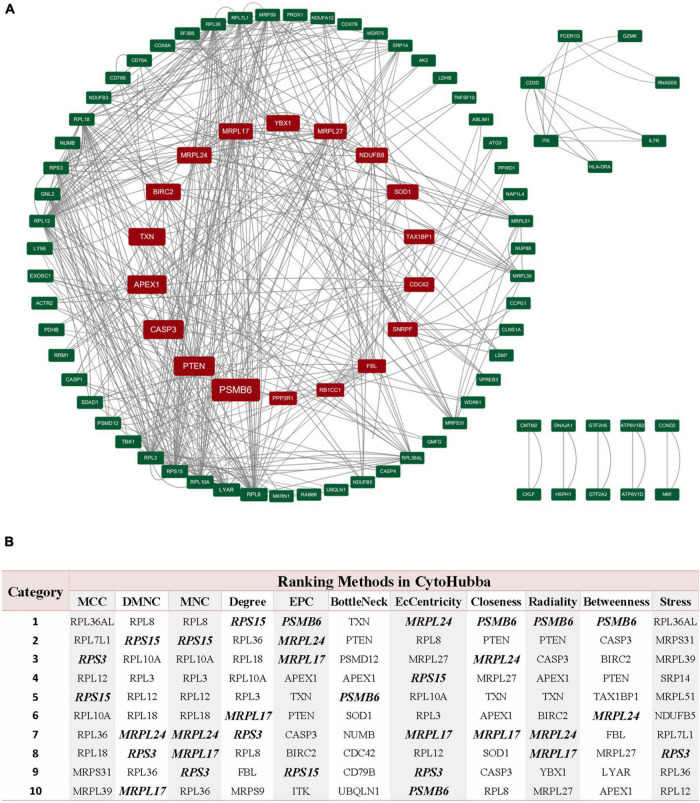
PPI network of common DEGs shared by AD and IS. **(A)** The circle nodes represent DEGs and edges represent the interactions between nodes. The PPI network has 65 nodes and 564 edges. **(B)** According to the 11 ranking methods provided by CytoHubba app, top 10 genes by at least 6 methods are referred as hub genes. Five hub genes were italicized and bold and the scores from all methods were labeled in table.

**FIGURE 5:**
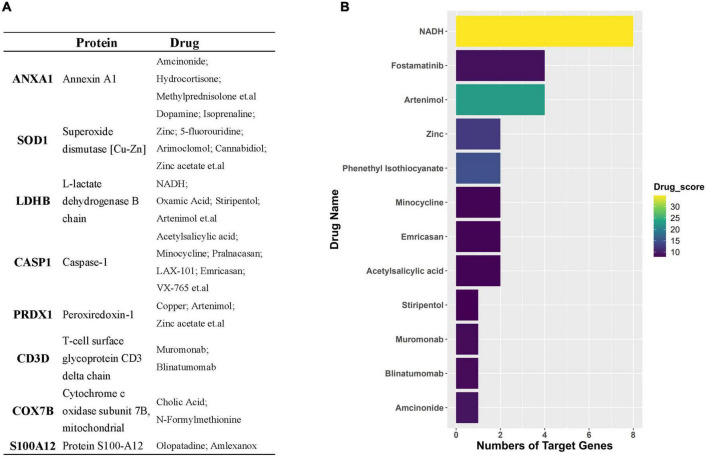
**(A)** Table represents target genes, corresponding proteins, and their potential drugs. **(B)** Bar plot demonstrates potential drugs, of which the horizontal axis represents the number of gene targets of the drug.

Through the PPI and network topology analysis for the common DEGs, we explored the hub genes that play indispensable roles in the shared biological mechanisms of AD and IS (Figure 4A). According to the eleven ranking methods provided by CytoHubba app to score these genes in the main PPI module, *RPS3*, *RPS15*, *PSMB6*, *MRPL17*, and *MRPL24* were identified as the hub genes (Figure 4B). These hub genes can be potential biomarkers, which may also provide new therapeutic targets. We further looked into whether there were drugs that can mitigate the process of gene expression differentiation (Supplementary Figure 4). By searching for interactions across three gene sets, DEGs of AD, DEGs of IS and DTGs, eight DEGs interacting with two known drug targets were identified, which were *ANXA1*, *SOD1*, *LDHB*, *CASP1*, *PRDX1*, *CD3D*, *NDUFB3*, and *TXN* (Figure 5A). The top ten drugs targeting these common DEGs were Fostamatinib, Artenimol, Zinc, Stiripentol, NADH, Phenethyl Isothiocyanate, Acetylsalicylic acid, Minocycline, Emricasan, and Amcinonide (Figure 5B). We validated these hub genes and drug targets with GSE37587, GSE46480, GSE140829 datasets, and results showed consistency (Supplementary Figure 5).

### Identification of regulatory transcript factors

**FIGURE 6:**
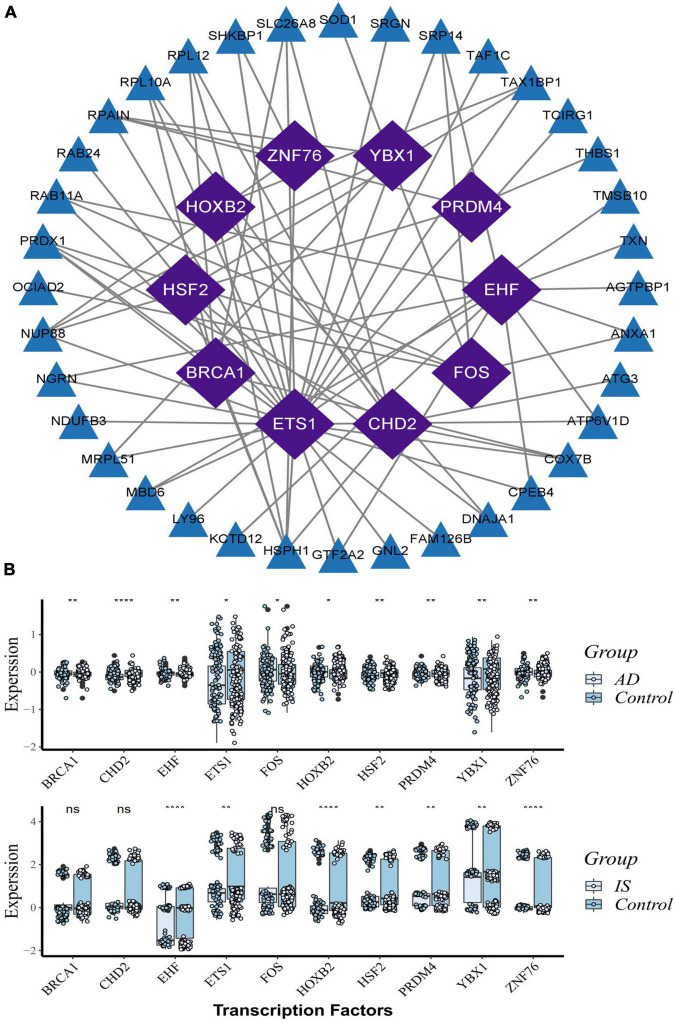
Transcription factors (TFs) regulatory network and their gene expression profiles in AD/IS. **(A)** TFs regulatory network. TFs were marked in purple, and the hub genes were marked in blue. **(B)** The gene expression level of TFs in AD and IS datasets. The comparison of gene expression between patients and controls was applied with *t*-test. *P*-value < 0.05 was considered statistically significant. AD, Alzheimer’s disease; IS, ischemic stroke. *P < 0.05; **P < 0.01; ****P < 0.0001.

Based on the RcisTarget package, we found ten possible TFs regulating the expression of these common DEGs (Figure 6A), 5 TFs of which were differentially expressed in the peripheral blood of AD and IS patients (Figure 6B). Among these TFs, *FOS* expression was up-regulation, while *PRDM4*, *HSF2*, *HOXB2*, and *ETS1* were down-regulation.

### Immune changes

**FIGURE 7:**
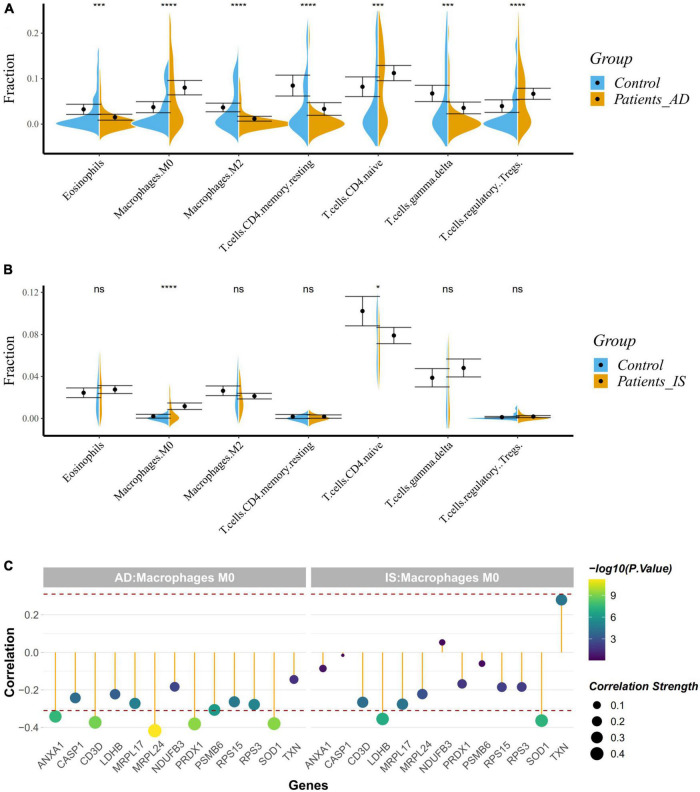
**(A,B)** Violin diagram showing the proportion of immune cells obtained using the CIBERSORT. *P < 0.05; ***P < 0.001; ****P < 0.0001. **(C)** Correlation between hub genes and drug target genes, and M0 macrophages. The red dashed lines represent +0.3 and –0.3.

Given the enrichment of the common DEGs on immune-related pathways, we applied the CIBERSORT classification algorithm on gene expression profiles to demonstrate changes of the immune system in AD and IS. The proportions of M2-type macrophages, M0-type macrophages, eosinophils, regulatory T cells, gamma-delta T cells, and CD4^+^ memory resting T cells, and CD4^+^ naive T cells were significantly different between AD patients and healthy controls (Figure 7A). Meanwhile, the proportions of M0-type macrophages and CD4^+^ naive T cells were also significantly different in IS cohort (Figure 7B), of which, M0-type macrophages were increased in AD and IS patients, and CD4^+^ naive T cells were increased in AD patients but decreased in IS patients.

Correlation analysis indicated a close relationship between hub genes, DTGs and M0-type macrophages (Figure 7C). *NUP88*, *CLNS1A*, *GTF2A2*, *ANXA1*, *SOD1*, *PRDX1*, and *CD3D* were negatively correlated with M0-type macrophages in AD patients (r < −0.3, *P* < 0.001), while *GTF2H5*, *SOD1*, and *LDHB* were negatively correlated with M0-type macrophages in the IS patients (r < −0.3, *P* < 0.001). Our analysis showed that *SOD1* was associated with the change of M0-type macrophages in both AD and IS patients.

## Discussion

Although large-scale GWASs of AD and IS have identified a set of risk loci and pleiotropic genes with genome-wide significance (Malik et al., 2018; Jansen et al., 2019), no shared genetic determinants between AD and IS have been reported (Traylor et al., 2016). Previous pathway-based association tests using large-scale GWAS summary datasets for AD and IS have found come common biological pathways shared by AD and IS (Cui et al., 2018). In this study, we analyzed the peripheral blood transcriptome of AD and IS patients using gene expression profile datasets from GEO (training datasets for IS: GSE16561 and GSE58294, for AD: GSE63060 and GSE63061; validation datasets for IS: GSE37587 and GSE46480, for AD: GSE140829) to search for supportive evidence for their relevance.

Through a comprehensive analysis, we revealed a total of 74 common DEGs shared by AD and IS. To ensure the biological meaning of the common DEGs, we randomly selected 600 genes from the expressed gene sets of AD and IS separately and repeated for 1,000 times (Supplementary Figure 6). The number of overlapped genes between AD and IS ranged from 6 to 32, obviously less than 74 (*t*-test, *P*-value < 0.001). GO and DO enrichment and GSEA analysis were further conducted for these common DEGs. For biological processes, the top GO terms were associated with immune system changes, such as T cell differentiation and activation, cell activation involved in immune responses, and mononuclear cell differentiation. For cellular components, ribosome, endocytic vesicle, ficolin-1-rich granule, mitochondrial protein-containing complex, and vesicle membrane were the top results. The structural constituent of ribosome is the only GO term which was affected by the common DEGs in molecular function experiment. The diseases enriched by DO analysis were mainly Alzheimer’s disease, tauopathy, atherosclerosis, and heart diseases. The results of GSEA suggested that cellular responses to stress and stimuli, innate immune System, translation, and neutrophil degranulation were the most significantly enriched pathways.

Therefore, the crucial mechanisms behind the correlation between AD and IS might focus on the immune system. As is known to all, a wide variety of immune cells exist in the brain and dysregulation of the innate immune system contribute to the onset and development of many neurological diseases, AD and IS included (Mastorakos and McGavern, 2019; Mezey et al., 2021). These results conform to previous studies. Cui et al. (2018) identified immunological processes in the shared biological pathways between AD and IS based on large-scale GWAS summary data. Other studies have also demonstrated the essential roles of immune system in AD and IS. Furthermore, we used CIBERSORT classification algorithm to conduct an immune cell enrichment analysis based on the merged datasets. We found that M0-type macrophages were both upregulated in AD and IS patients, however, CD4+ naive T cells were upregulated in AD patients but down-regulated in IS patient. Our results agree with those of Liu C. et al. (2022) and Wang X. et al. (2022), which showed that resting CD4 T memory cells were significantly down-regulated whereas M0 macrophages were significantly up-regulated in IS. This indicated a putative relationship between immune system and IS. Recent studies have elucidated that M2 macrophages, CD4 naive T cells, regulatory T cells, eosinophils, gamma delta T cells, resting mast cells, M0 macrophages and activated CD4 memory T cells are closely correlated with AD, which further verified our discovery. Correlation analyses confirmed a strong relationship between hub genes, DTGs, and M0-type macrophages. This study identified a common gene, *SOD1* to be negatively correlated with M0-type macrophages in AD and IS patients, suggesting its potential role in the shared immune changes of AD and IS.

Further PPI network analysis was constructed to identify the most significant clusters of DEGs and understand the biological characteristics of the proteins. Here, we identified five hub genes based on topological measures that might suggest common pathogenesis behind AD and IS. Both *RPS3* and *RPS15* encode a ribosomal protein, which is part of the 40S subunit. RPS3 induces neuronal apoptosis by interacting with the E2F1 TF and inducing the expression of pro-apoptotic proteins BCL2L11/BIM and HRK/Dp5 (Lee et al., 2010), while the phosphorylation of *RPS15* is related to LRRK2 neurodegeneration and neurotoxicity (Martin et al., 2014). Wu et al. (2022) suggested that RPS3 and RPS15 may be potential targets and treatment for early diagnosis of AIS. *PSMB6*, also known as 20S proteasome subunit beta-1, codes for the β1 core catalytic subunit of the proteasome (Vriend and Marzban, 2017). A study showed that PSMB6 was a critical regulator of circadian rhythm, which may also have a direct or indirect effect on neurodegenerative diseases (Beker and KiliÇ, 2020). Mitochondrial ribosomal protein large 17 (MRPL17) and Mitochondrial ribosomal protein large 24 (MRPL24) are one of the 82 protein components of mitochondrial ribosomes, playing an essential role in the mitochondrial translation process, but their relationship with neurodegenerative diseases is currently unclear (Nottia et al., 2020). In addition, we found 10 possible TFs regulating the expression of these genes. By further verification, we found that five TFs are differentially expressed in AD and IS, including FOS, PRDM4, HSF2, HOXB2, and ETS1. They coordinately participated in the regulation of two hub genes (*PSMB6*, *RPL17*).

We next detected the candidate drugs for AD and IS based on the intersection across three gene sets, DEGs_AD, DEGs_IS, and DTGs. Here, we identified eight DEGs, including *ANXA1*, *SOD1*, *LDHB*, *CASP1*, *PRDX1*, *CD3D*, *NDUFB3*, and *TXN*. Overwhelming evidence has confirmed the protective role of ANXA1 in neuronal apoptosis during cerebral ischemia (Zhao et al., 2015; Li et al., 2019). Recently, Miriam Ries et al. discovered that ANXA1 could restore cerebrovascular integrity and reduce amyloid-β and tau. Moreover, ANXA1 has been reported to have therapeutic potential in ischemia-reperfusion injury (Ansari et al., 2018) and protecting against the breakdown of the blood-brain barrier in AD (Park et al., 2017). *SOD1* has previously been reported to correlate with neurodegenerative diseases, such as amyotrophic lateral sclerosis (Renton et al., 2014) and AD (Bader et al., 2020). Many studies have demonstrated that *CASP1* may be a therapeutic target against cognitive impairment and inflammation in AD (Kaushal et al., 2015; Gu et al., 2021; Flores et al., 2022). In addition, the inhibition of *CASP1* has proven to relieve cerebral ischemia in a murine model by targeting the canonical inflammasome pathway of pyroptosis that is important for neuronal death in acute IS (Li et al., 2020). Drugs targeting these genes include Fostamatinib, Artenimol, NADH, Phenethyl Isothiocyanate, Acetylsalicylic acid, Minocycline, Emricasan, and Amcinonide. The recent study has confirmed NADH can not only improve cellular energy metabolism after IS, but also can inhibit oxidative stress by decomposing into NAD^+^, protect mitochondrial function, and reduce cerebral ischemia-reperfusion injury (Wang X. X. et al., 2022). As the NAD^+^ donor, NADH further appeared as a protective agent for AD because of the indispensable role of NAD^+^ depletion and impairment of NAD^+^-dependent pathways in AD pathophysiology (Lautrup et al., 2019). Acetylsalicylic acid, also known as aspirin, an anti-inflammatory medication, has been verified to be useful in prevention cognitive deterioration due to its anti-thrombotic and anti-inflammatory properties. It has shown promising performances for treating IS and AD in multiple pre-clinical and clinical trials (Wang et al., 2013; Johnston et al., 2018, 2020; Weng et al., 2021). Minocycline, an antibiotic, has been proven to have neuroprotective effects in AD and IS. Studies have reported that Minocycline could not only mitigate Alzheimer’s-like pathology and improve cognition, but also exhibit similar promise in the treatment of IS when administered alone or in combination with thrombolyticse (Campbell et al., 2011; Fagan et al., 2011). Emricasan is a caspase inhibitor and is currently used for several liver diseases in clinical trials (Shiffman et al., 2010). The findings of Tian et al. (2018) firstly lay a basis for the use of emricasan to treat IS.

This study has the following limitations. Firstly, this study was conducted basing on bioinformatic and correlational analyses, and differences in microarray platforms, blood collection, and RNA extraction methods, statistical methods could produce potential bias for the results. Besides, the size of the datasets used in this study might not be large enough to generate very powerful results. More large cohorts of AD, IS patients are needed, and future cellular or animal experiments are expected to provide convincing proofs for our results. Therefore, the above findings should be taken with carefulness. Nevertheless, this study provides new insights into the shared pathogenesis behind AD and IS, suggesting the important role of immune changes and several promising genes for the onset and development of these two diseases.

## Data availability statement

The datasets presented in this study can be found in online repositories. The names of the repository/repositories and accession number(s) can be found in the article/Supplementary material.

## Author contributions

WL: methodology, software, investigation, visualization, and writing—original draft. MW: methodology, validation, and writing—review and editing. YS: investigation and data curation. X-ZY: writing—review and editing, supervision, project administration, and funding acquisition. All authors contributed to the article and approved the submitted version.

## Footnotes

1
http://www.ncbi.nlm.nih.gov/geo/

2
http://www.genome.jp/kegg/

3
https://apps.cytoscape.org/apps/cytohubba

4
https://go.drugbank.com/releases/latest
