# Supplementary material for: Transcriptomic analysis identifies shared biological foundations between ischemic stroke and Alzheimer’s disease
Source: Front Neurosci. 2022 Nov 18;16:1008752. doi: 10.3389/fnins.2022.1008752 (PMC9715755; doi:10.3389/fnins.2022.1008752)
Supplement: Supplementary file 1 [file Data_Sheet_1.docx]

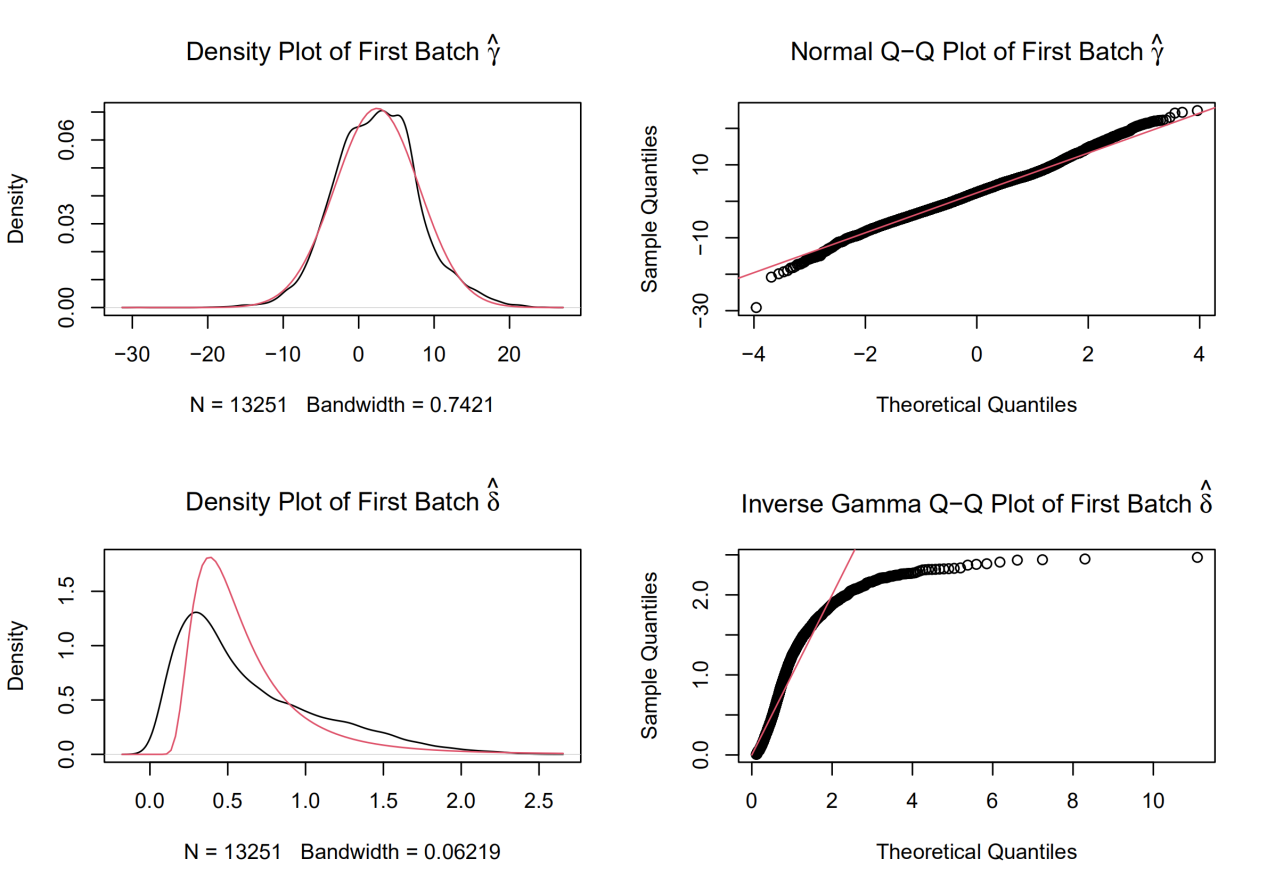
**Supplement Figure 1.** Quality control for expression data matrix. The density plot intuitively shows the probability distribution of the samples’ gene expression after removing the batch-effect. The quantile-quantile (Q-Q) plot examined whether the gene expression of samples obey the normal distribution after quality control.


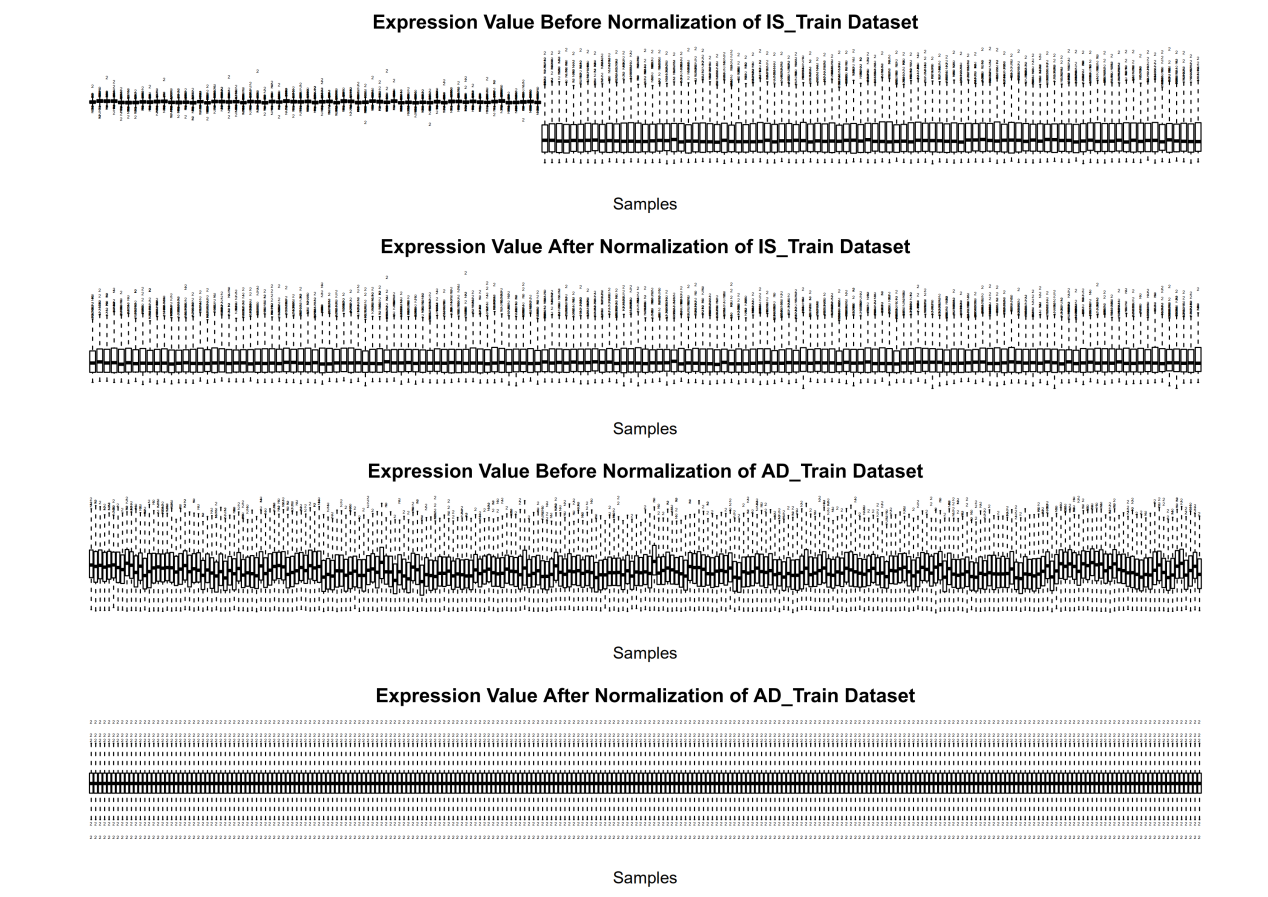


**Supplement Figure 2.** Boxplot of samples’ gene expression. The boxplot clearly shows the changes of probability distribution after normalization. The X-axis is the sample number, and Y-axis is the expression level.


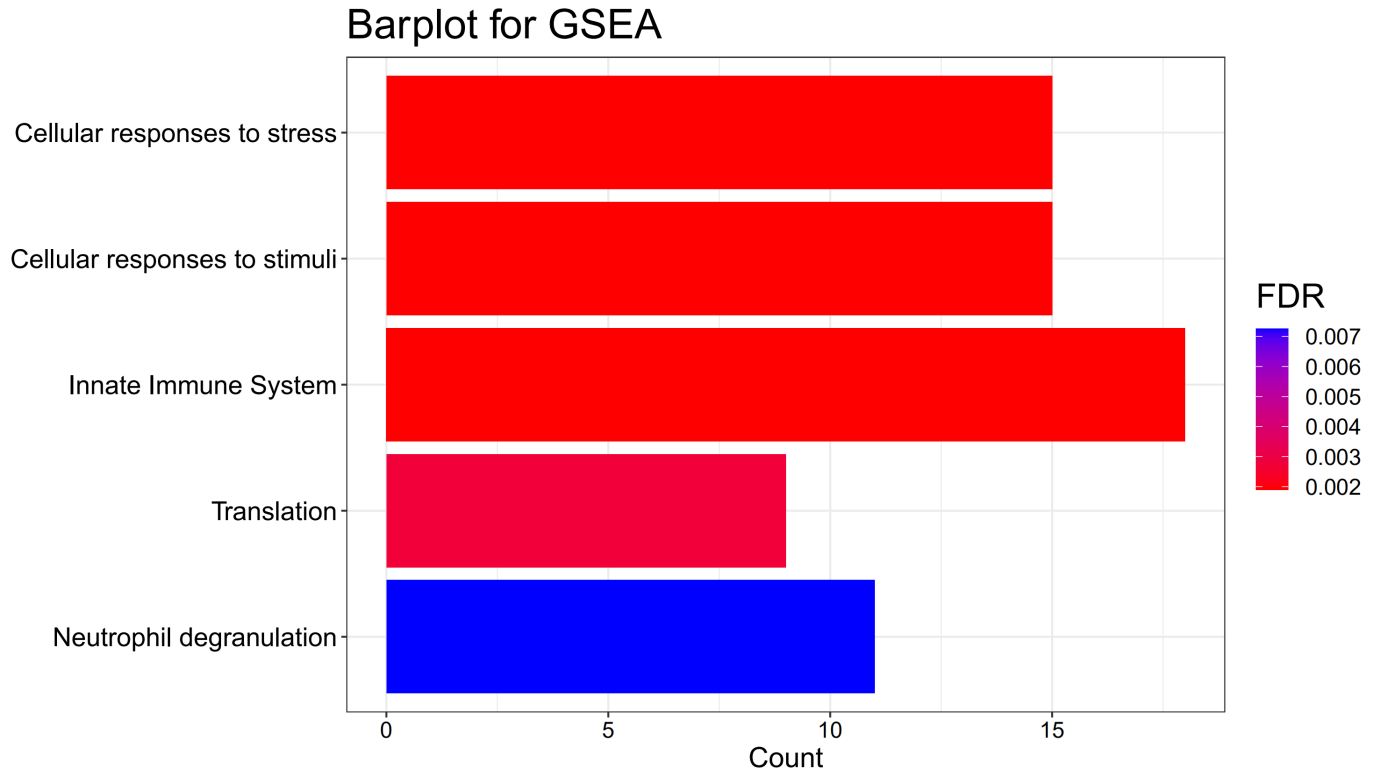
**Supplement Figure 3.** Gene set enrichment analysis, where the horizontal axis represents the number of DEGs under the gene set enrichment analysis (GSEA) term.


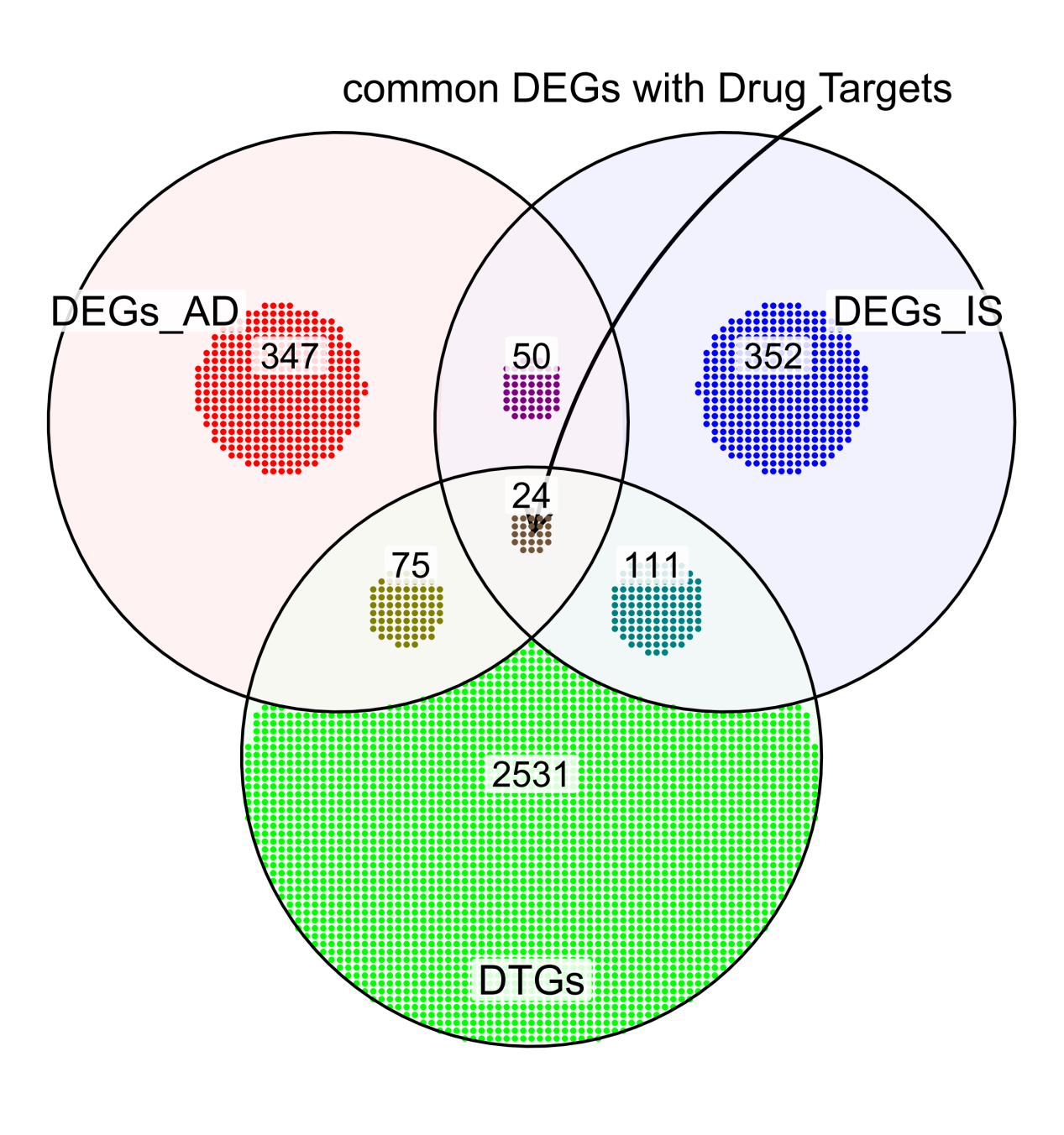


**Supplement Figure 4.** Venn diagram shows the target genes of potential drugs obtained from the three gene sets.


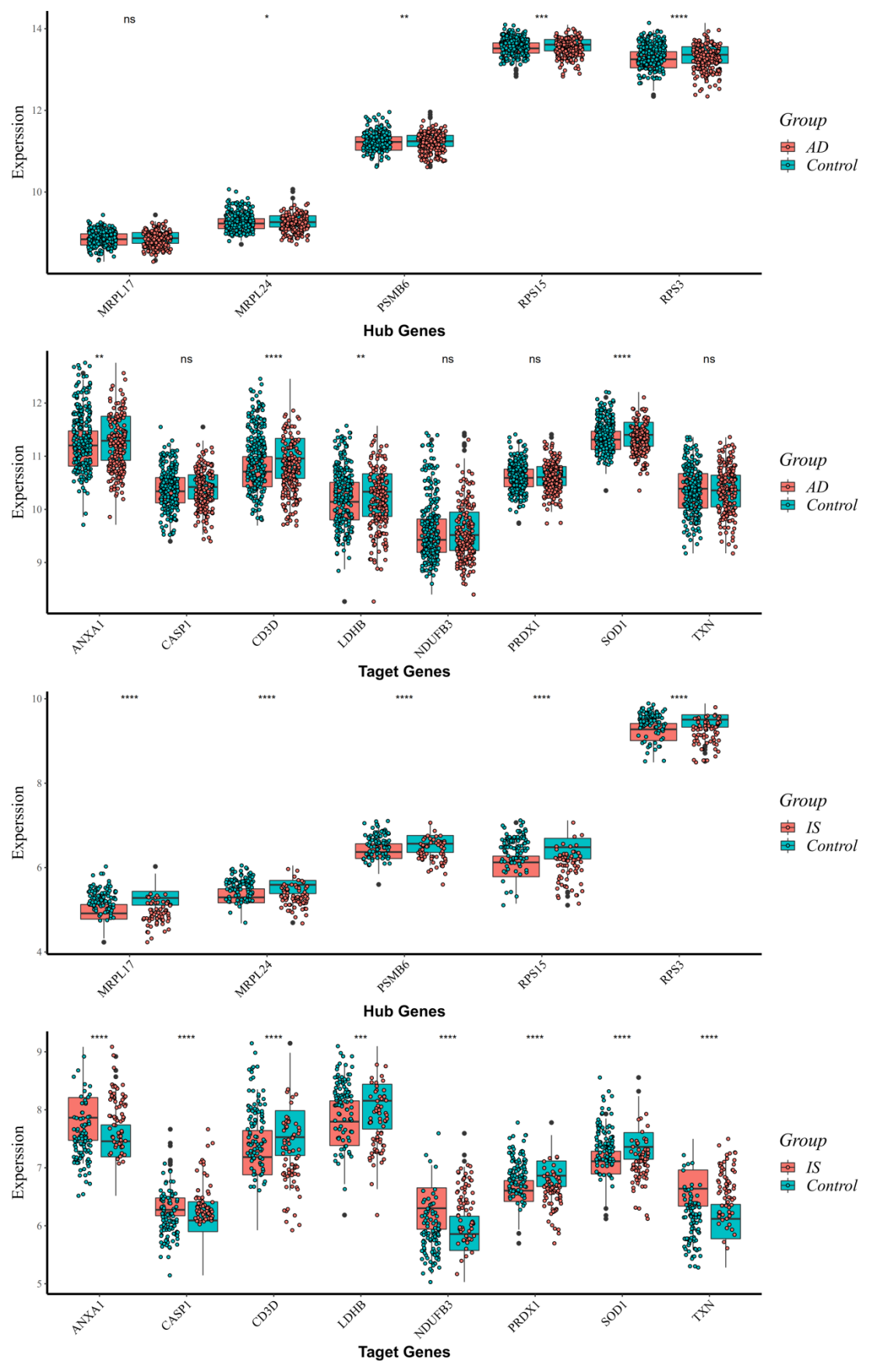
**Supplement Figure 5.** Gene expression level of hub genes and drug target genes in AD/IS patients and healthy controls in validated datasets. *:P < 0.05; **:P < 0.01; ***:P < 0.001; ****:P < 0.0001


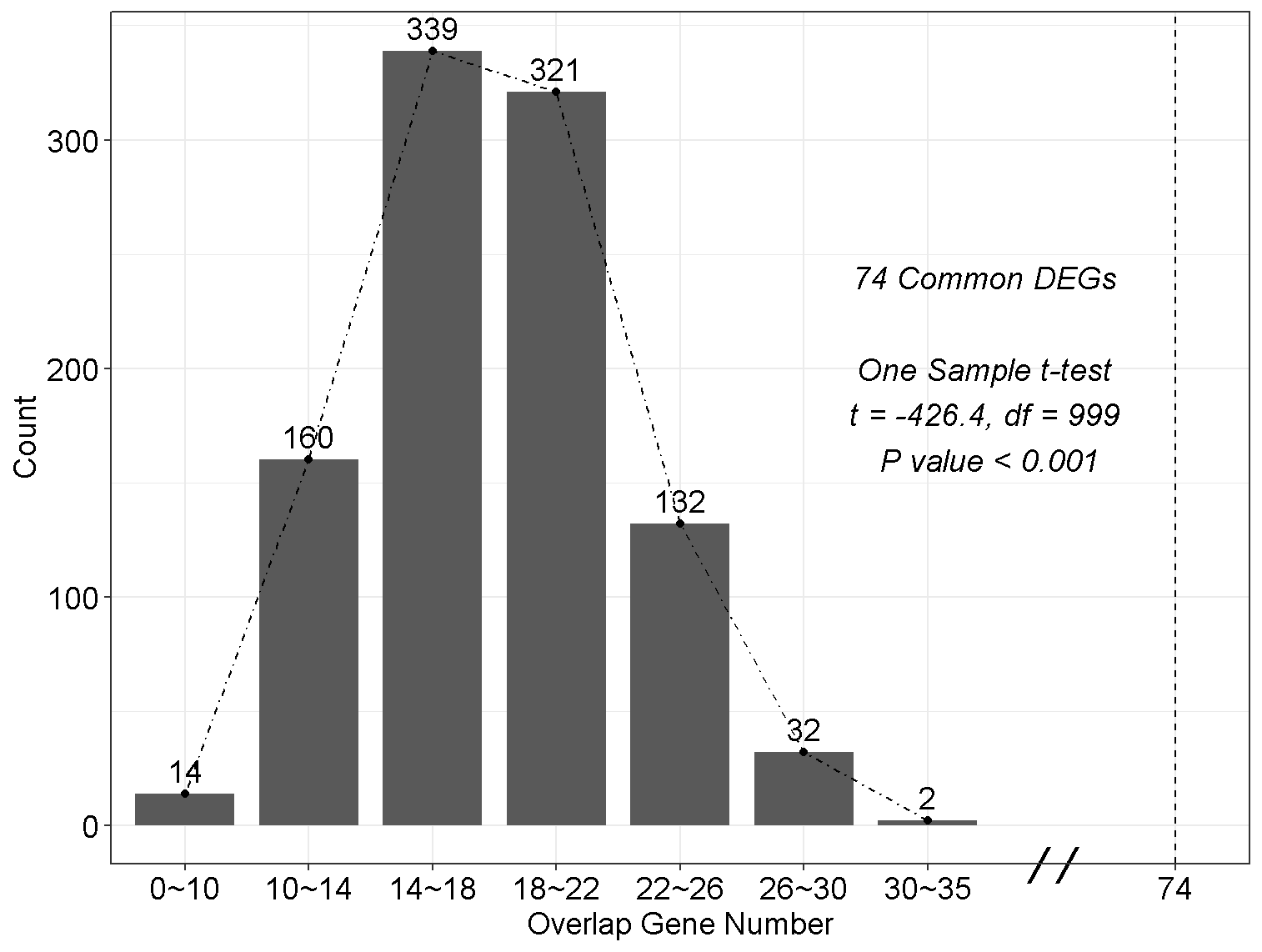


**Supplement Figure 6.** Distribution of gene number overlapped between AD and IS. 600 genes were randomly selected from AD and IS separately and interaction were permutated for 1000 times. The real data was represented with the dotted line.
